# Riedel thyroiditis secondary to papillary thyroid carcinoma: A case report

**DOI:** 10.1097/MD.0000000000047649

**Published:** 2026-02-13

**Authors:** Tongquan Zhao, Shuqin Wang, Min Zhang

**Affiliations:** a Department of Surgery, People’s Hospital of Rizhao City, Rizhao, China; b Department of Gynaecology and Obstetrics, People’s Hospital of Rizhao City, Rizhao, China; c Department of Ultrasound, People’s Hospital of Rizhao City, Rizhao, China.

**Keywords:** IgG4-related disease, malignant tumors, Riedel thyroiditis

## Abstract

**Rationale:**

Riedel thyroiditis (RT) is categorized as an immunoglobulin G4-related disease (IgG4-RD), a condition that is seldom encountered in clinical practice. IgG4-RDs have been linked to an elevated risk of malignancy. Although prior studies have documented instances where IgG4-RDs were induced by malignant tumors, such occurrences are still rarely reported in both the domestic and international literature.

**Patient concerns:**

A 47-year-old male, asymptomatic except for a palpable right neck nodule detected during routine follow-up, was diagnosed with RT secondary to papillary thyroid carcinoma. To our knowledge, this is the inaugural case of its nature ever documented, providing valuable insights into the relationship between malignant tumors and IgG4-RDs.

**Diagnoses:**

Histopathological examination revealed fibrous tissue hyperplasia, atrophic thyroid follicles, extensive infiltration of lymphocytes and plasma cells, and perivascular inflammation. The pathological diagnosis was consistent with RT.

**Interventions:**

The patient underwent thyroidectomy and neck lymph node dissection.

**Outcomes:**

The patient recovered uneventfully, takes levothyroxine daily to suppress thyroid-stimulating hormone, and undergoes a thyroid ultrasound and thyroid function test every 6 months.

**Lessons:**

RT can mimic malignancy on ultrasound (e.g., thyroid imaging reporting and data system 5 features). Greater awareness and integrated clinicopathologic evaluation may prevent unnecessary surgery.

## 1. Introduction

Riedel thyroiditis (RT), also known as chronic invasive fibrous thyroiditis, is an uncommon clinical condition, occurring at a rate of roughly 1.06 cases per 100,000 individuals. It primarily impacts women between the ages of 30 and 50 years, with females being affected about 3 times more frequently than males.^[1]^ Recent studies^[2]^ have identified immunoglobulin G4 (IgG4)-positive plasma cells in RT, leading to its classification within the spectrum of IgG4-related diseases (IgG4-RDs). IgG4-related thyroid disease is categorized into 4 subtypes, one of which is RT.^[3]^ Although RT falls under the category of IgG4-RD, the presence of serum IgG4 elevation and IgG4-positive plasma cells in pathological tissues is not included in the diagnostic criteria for RT.^[4]^

The literature has reported that malignant tumors often occur concurrently with IgG4-RD, suggesting a potential association between the two; however, further research is needed. Accumulating evidence suggests a close relationship between IgG4-RD patients and malignant tumors. Yamamoto et al^[5]^ observed the development of malignant tumors in 11 (10.4%) of 106 IgG4-RD patients during follow-up. These malignant tumors varied, including lung cancer, colon cancer, and lymphoma. In a study by Bojková et al on 11 pancreatic cancer patients, 7 patients had IgG4 levels twice the normal range (65.6%), and these individuals were diagnosed with autoimmune pancreatitis. This further confirms the correlation between the two.^[6]^ A case report describes a 47-year-old male patient who developed papillary thyroid carcinoma (PTC) and subsequently presented with RT 1 year postsurgery.

## 2. Case presentation

**Figure 1.**
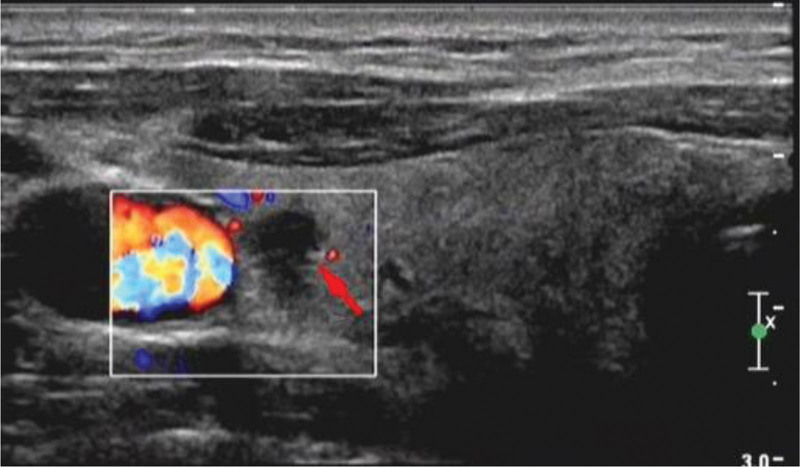
Color Doppler ultrasound of the right thyroid lobe shows a solid hypoechoic nodule (red arrow) in the upper pole with irregular margins, taller-than-wide shape, absence of hypoechoic halo, and scant intralesional vascularity. The lesion was classified as TI-RADS 5. TI-RADS 5 = thyroid imaging reporting and data system 5.

A 47-year-old male patient presented with the discovery of nodules in the right lobe of the thyroid gland, which had been noted for 1 month. The patient reported no compressive symptoms such as dysphagia, hoarseness, or dyspnea. The patient had undergone left lobe and isthmus thyroidectomy for PTC 1 year prior and had been under routine biannual follow-up with high-resolution neck ultrasound and thyroid function tests. The patient had been receiving levothyroxine 100 µg daily for thyroid-stimulating hormone suppression since the previous thyroid surgery. Thyroid ultrasound revealed solid hypoechoic nodules measuring ~0.75 × 0.51 × 0.67 cm in the upper pole of the right lobe, characterized by indistinct margins, absence of a hypoechoic halo, an aspect ratio >1, and minimal blood flow signals (Fig. 1). Ultrasound demonstrated thyroid imaging reporting and data system 5 (TI-RADS 5) features: solid hypoechoic nodule, irregular margins, taller-than-wide shape, absence of hypoechoic halo, and scant internal vascularity. Based on TI-RADS 5 features, differentials included recurrent PTC, follicular variant PTC, Hashimoto thyroiditis with fibrotic change, and RT. To establish a definitive diagnosis, a right thyroid lobectomy was performed under general anesthesia. Preoperative laboratory tests showed free thyroxine3 4.89 pmol/L (2.43–6.01), free thyroxine4 15.21 pmol/L (9.01–19.05), thyroid-stimulating hormone 1.068 mIU/L (0.35–4.94), thyroglobulin <0.2 ng/mL, erythrocyte sedimentation rate 9 mm/h, and C-reactive protein 3.2 mg/L, with vital signs such as blood pressure 126/78 mm Hg, heart rate 76/min, and temperature 36.7°C. Serum IgG4 was not obtained preoperatively.

**Figure 2.**
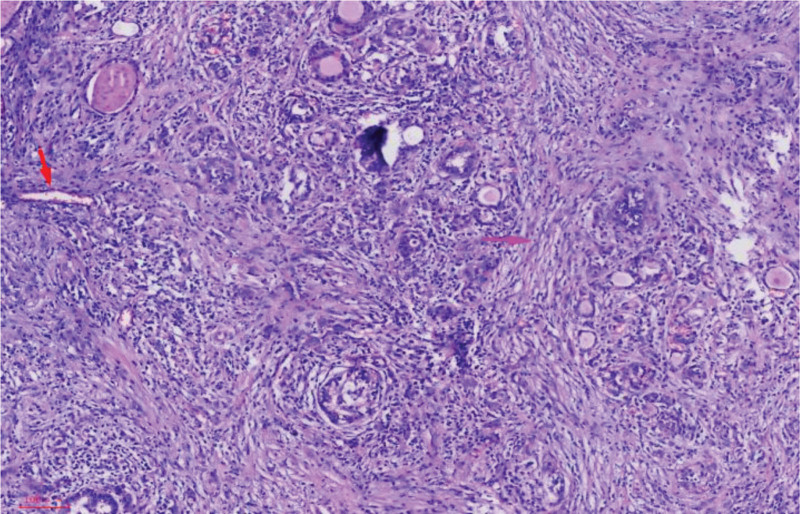
Hematoxylin–eosin staining at ×20 demonstrates dense hyaline fibrosis and atrophic thyroid follicles. The red arrow indicates occlusive phlebitis; the pink arrow highlights dense fibrosis. There are no malignant epithelial cells, granulomas, or giant cells.

The diagnosis of RT was made based on surgical histopathology showing dense hyalinized fibrosis, atrophic thyroid follicles, lymphoplasmacytic infiltration, perivascular inflammation, and occlusive phlebitis (Fig. 2). Immunostaining was not performed as histopathological features were diagnostic. Considering the patient’s medical history, it is plausible that the RT could be a secondary manifestation of PTC. Written informed consent for publication of the clinical details and images was obtained from the patient prior to reporting this case. This manuscript has been approved by the Ethics Committee of Rizhao People’s Hospital, Opinion No.: 2025-MR-93-01.

## 3. Discussion

RT is pathologically characterized by extensive fibroplasia with significant infiltration of lymphocytes and plasma cells. This fibrosis often extends beyond the thyroid capsule, infiltrating adjacent tissues and structures, which results in a range of compressive symptoms including dysphagia, dyspnea, hoarseness, and others.^[7,8]^ Thyroid function tests and inflammatory markers may assist in evaluation, but the results can be normal in RT, as in our patient. Serum IgG4 can be elevated in IgG4-RD, but it is not required for RT diagnosis. Histopathology remains the definitive diagnostic method. The case fulfills RT diagnostic criteria: dense hyalinized fibrosis with extrathyroidal extension, occlusive phlebitis, absence of granulomas/giant cells/lymphoid follicles, and absence of malignancy at the RT site. Differential diagnoses included infiltrative PTC, fibrosing Hashimoto thyroiditis, and anaplastic carcinoma. Infiltrative PTC typically displays papillary architecture, nuclear grooves, and psammoma bodies; these were absent. Fibrosing Hashimoto shows lymphoid follicles and Hürthle cells without occlusive phlebitis. Anaplastic carcinoma presents pleomorphic malignant cells, high mitotic activity, and necrosis, also absent here.

Since the discovery of IgG4-RD, the relationship between these conditions and malignant tumors has emerged as a focal point of research. Studies have shown that cytokines play a pivotal role in immune responses, chronic inflammatory stimulation, and potentially in the induction of malignant tumors, thereby increasing the risk of malignancy by 2.7-fold compared with the general population.^[9]^ Sumimoto et al found that the estimated overall prevalence of malignancies in IgG4-RD cases was 900 per 100,000, which is significantly higher than that in the general population. In the data of 200 patients, 30.5% of cancer cases were identified 2 years or more before the diagnosis of IgG4-RD, 46% were found within 1 year before or after the diagnosis, and 31% were identified 2 years or more after the diagnosis.^[10]^ These findings reveal that IgG4‑RD patients with prior malignancy tend to be older, have shorter latency between cancer diagnosis and IgG4‑RD onset, and display different organ involvement profiles compared with those without malignancy history, suggesting potential pathophysiologic differences. A study conducted a controlled analysis of 125 patients with IgG4-RD and revealed that the prevalence of prior malignant tumor history is 3-fold higher than that in the general population and none of the observed cases developed at the site of the previous malignancy. This increased incidence may be attributed to alterations in the immune microenvironment induced by cancer treatments or subsequent tissue damage, or it could result from new antigens exposed by tumor gene mutations.^[11]^ RT following PTC may involve immune‑mediated fibrotic pathways, including chronic antigenic stimulation from tumor remnants, postsurgical tissue remodeling, and cytokine‑driven fibroblast activation (e.g., transforming growth factor-beta). Recruitment of IgG4‑positive plasma cells and skewing toward Th2/Treg immune responses may promote dense fibrosis in predisposed individuals.

Herein, we present the first documented case of RT secondary to PTC both domestically and internationally, as confirmed by surgical intervention. To our knowledge, there are no similar documented cases in the literature. RT can be treated medically with glucocorticoids, which may reduce fibrosis and inflammation, and tamoxifen, which has antifibrotic effects. Surgery may be considered for diagnosis or to relieve compressive symptoms. This particular case of RT occurring secondary to a primary malignant tumor provides significant insights into the localization of the disease and highlights the urgent need for additional research in this field. Mechanistically, shared pathways involving chronic antigenic stimulation, Th2/Treg skewing, cytokine signaling, and recruitment of IgG4-positive plasma cells may drive fibroblast activation and hyalinized fibrosis in susceptible hosts. Diagnostic integration is essential because RT frequently mimics malignancy on imaging. When TI-RADS 5 features occur in a patient with prior PTC, decision-making should weigh fine needle aspiration feasibility (often limited by dense fibrosis) against the need for surgical exploration to obtain definitive histology. This case highlights the critical need for enhanced clinical awareness of RT, which is often underrecognized and typically diagnosed postoperatively through pathological examination. Improving understanding of this rare condition can help prevent unnecessary surgeries and reduce patient burden. Currently, diagnostic criteria for RT lack standardization, and the relationship between RT and malignancies remains poorly understood. Further research with a larger accumulation of cases is essential to advance our knowledge.

## Author contributions

**Conceptualization:**Tongquan Zhao.

**Methodology:** Tongquan Zhao.

**Resources:** Shuqin Wang, Min Zhang.

**Data curation:** Shuqin Wang, Min Zhang.

**Investigation:** Shuqin Wang, Min Zhang.

**Writing – original draft:** Tongquan Zhao.

**Writing – review & editing:** Min Zhang.
